# Retraction Note: Repertoire of the gut microbiota from stomach to colon using culturomics and next-generation sequencing

**DOI:** 10.1186/s12866-023-03167-3

**Published:** 2024-01-03

**Authors:** Morgane Mailhe, Davide Ricaboni, Véronique Vitton, Jean-Michel Gonzalez, Dipankar Bachar, Grégory Dubourg, Frédéric Cadoret, Catherine Rober, Jérémy Delerce, Anthony Levasseur, Pierre-Edouard Fournier, Emmanouil Angelakis, Jean-Christophe Lagier, Didier Raoult

**Affiliations:** 1 Aix Marseille Univ, IRD, MEPHI, IHU - Méditerranée Infection, 19-21 Boulevard Jean Moulin, Marseille, 13005 France; 2 Department of Clinical and Biomedical Sciences Luigi Sacco, III Division of Infectious Diseases, University of Milano, Via GB Grassi, 74, Milan, 20157 Italy; 3 Service de Gastroentérologie, Hôpital Nord, Assistance Publique-Hôpitaux de Marseille, Marseille, 13915 France; 4 Aix Marseille Univ, IRD, VITROME, IHU - Méditerranée Infection, 19-21 Boulevard Jean Moulin, Marseillle, 13005 France

**Retraction note: BMC Microbiol 18, 157 (2018)**

10.1186/s12866-018-1304-7

The Editor has retracted this article because the authors could not provide evidence of approval from an appropriate ethics committee. Grégory Dubourg, Jean-Christophe Lagier, and Didier Raoult disagree with this retraction. The remaining authors did not respond to correspondence from the publisher about this retraction.

